# The Tree Drought Emission MONitor (Tree DEMON), an innovative system for assessing biogenic volatile organic compounds emission from plants

**DOI:** 10.1186/s13007-017-0166-6

**Published:** 2017-03-20

**Authors:** Marvin Lüpke, Rainer Steinbrecher, Michael Leuchner, Annette Menzel

**Affiliations:** 1 Ecoclimatology, Technische Universität München, Hans-Carl-von-Carlowitz-Platz 2, 85354 Freising, Germany; 2 TUM Institute for Advanced Study, Lichtenbergstraße 2 a, 85748 Garching, Germany; 3 Department of Atmospheric Environmental Research (IMK-IFU), Institute of Meteorology and Climate Research, Karlsruhe Institute of Technology (KIT), Kreuzeckbahnstraße 19, 82467 Garmisch-Partenkirchen, Germany; 4 Springer Science+Business Media B.V., Van Godewijckstraat 30, 3311 GX Dordrecht, The Netherlands

**Keywords:** Dynamic chambers, BVOC, Drought, Monoterpene, *Castanea sativa* Mill., Sweet chestnut

## Abstract

**Background:**

Biogenic volatile organic compounds (BVOC) emitted by plants play an important role for ecological and physiological processes, for example as response to stressors. These emitted compounds are involved in chemical processes within the atmosphere and contribute to the formation of aerosols and ozone. Direct measurement of BVOC emissions requires a specialized sample system in order to obtain repeatable and comparable results. These systems need to be constructed carefully since BVOC measurements may be disturbed by several side effects, e.g., due to wrong material selection and lacking system stability.

**Results:**

In order to assess BVOC emission rates, a four plant chamber system was constructed, implemented and throughout evaluated by synthetic tests and in two case studies on 3-year-old sweet chestnut seedlings. Synthetic system test showed a stable sampling with good repeatability and low memory effects. The first case study demonstrated the capability of the system to screen multiple trees within a few days and revealed three different emission patterns of sweet chestnut trees. The second case study comprised an application of drought stress on two seedlings compared to two in parallel assessed seedlings of a control. Here, a clear reduction of BVOC emissions during drought stress was observed.

**Conclusion:**

The developed system allows assessing BVOC as well as CO_2_ and water vapor gas exchange of four tree specimens automatically and in parallel with repeatable results. A canopy volume of 30 l can be investigated, which constitutes in case of tree seedlings the whole canopy. Longer lasting experiments of e.g., 1–3 weeks can be performed easily without any significant plant interference.

**Electronic supplementary material:**

The online version of this article (doi:10.1186/s13007-017-0166-6) contains supplementary material, which is available to authorized users.

## Background

Biogenic volatile organic compounds (BVOC) are emitted by the biosphere. The annual global flux of BVOC of 1.091 Gt a^−1^ for the year 2000 is estimated to consist of 49% isoprene, 14% monoterpene and 35% of various other volatile organic compounds (VOC) [1]. One major source of BVOC is the biochemical synthesis within plants; BVOC are then either stored or emitted directly [2]. Depending on the latter pathways BVOC emissions are strongly driven by light and/or temperature [3].

The production and emission of BVOC by plants is linked to a wide range of ecological functions, such as response to herbivore feeding by attracting potential predators or acting as repellent [4–7]; communication processes among plants or between plants and insects [8], e.g., BVOC related to herbivory induce the production of defense substances in non-attacked specimens [7, 9]; and attraction of pollinators to open flowers [5]. For the plant itself BVOC seem to reduce oxidative stress in case of heat waves or high ozone concentrations [10] and other stress induced by the complex abiotic urban environment [11].

Beside their ecological functions, BVOC play a significant role in atmospheric chemistry [12], such as in formation of biogenic secondary organic aerosols (bSOA) [13, 14]; in O_3_ formation in the presence of NO_x_ [15] a well as in O_3_ destruction and OH reduction and production [16]. These processes can contribute to environmental pollution [17], thus influencing the global climate [18]. Oxidation of BVOC in the atmosphere may result in positive or negative feedbacks on the plants themselves and their BVOC production [19].

In order to model BVOC fluxes for different ecosystems [20–22] experimental data on the ecosystem-, tree- and leaf-level for parameterization and validation as well as a deeper process understanding are needed. BVOC fluxes at ecosystem-level are typically derived by micro-meteorological measurement techniques [23–29], whereas at plant- and leaf-level chamber/enclosure measurements [30–36] are used. Several excellent review articles [37–40] describe the relevant specifications and requirements for reproducible and accurate chamber experiments as well as potential sources of error. Ortega and Helmig [38] also gives a comprehensive overview on previously performed enclosure measurements. In general a dynamic chamber design with constant air exchange (mass flow controlled) is preferred, since this design may reach steady state conditions fast and consequently the built up of water vapor and extreme chamber heat is reduced [37–40]. Both factors are disadvantageous: water condensation in the chamber system would lead to compound losses and extreme heat would introduce stress for the plant [39], e.g., indicated by reduced transpiration and photosynthesis. Depending on the experiment location and design, regulation of temperature, CO_2_ concentration and water vapor at inlets as well as illumination control should be considered. Thus, an effective and fast control of the environmental conditions for plants studied is desirable for achieving faster steady state conditions and thus stable gas exchange (see e.g., [41, 42]). In order to reduce wall losses or on-wall-reactions, inert materials should be used for constructing such a gas exchange study system, e.g., fluorinated plastics or stainless steel. In addition, a careful, fast, and accurate monitoring of the chamber environment and the plant status is needed for an exact quantification of leaf to air gas exchange.

The reported technical solutions range from simple branch bags [35, 43] to environmentally controlled inert chambers [36, 44–48]. Most studies use either commercial leaf chamber systems [32, 49, 50] or self-build chambers [44], yet multiple parallel (N > 2) chamber designs are rarely presented [51, 52]. Intensive BVOC screening studies or treatment-effect studies (e.g., stress vs. control), however, would benefit from a greater number of simultaneously operated measurement chambers allowing larger sample sizes at a time or direct comparisons, respectively, and thus minimizing the number of (distracting) co-variables (e.g., growth or phenological development).

In order to investigate gas exchange of small trees under different environmental conditions and for different physiological states, the dynamic enclosure system Tree Drought Emission MONitor (Tree DEMON) was developed and evaluated. Using Tree DEMON BVOC emissions of four potted trees with a crown volume of up to 30 l were measured in parallel. Additionally, CO_2_ and water vapor gas exchange as well as environmental parameters, such as air temperature, light, soil moisture, are monitored and controlled with an integrated data acquisition and control system. The focus of this study lies on the Tree DEMON development, its rigid performance testing and two case studies on BVOC emissions of sweet chestnut (*Castanea sativa* Mill.) demonstrating the power of the whole system for plant ecophysiological experiments.

## Materials and methods

### Tree DEMON system

#### System layout

**Fig. 1:**
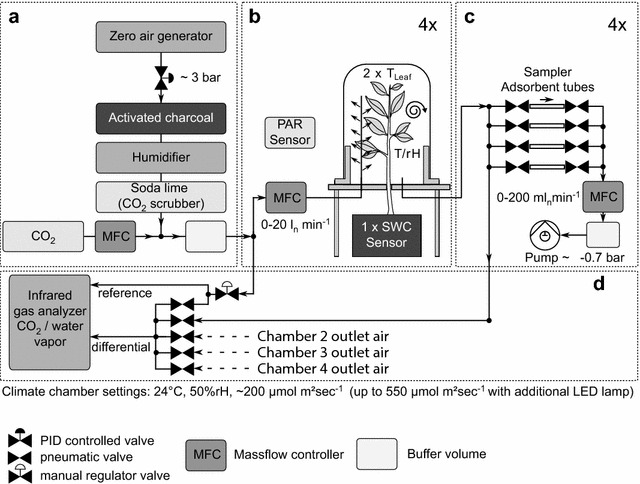
System schematic. The Tree DEMON gas exchange study system design. **a** Purge air supply and conditioning; **b** chambers with sensors; **c** BVOC sampler unit containing four sampler strings with four ports each; **d** CO_2_ and water vapor gas exchange unit. *Arrows* indicate the direction of air flow. *MFC* mass flow controller, *T/rH* temperature and relative humidity sensor, *T*_*leaf*_ leaf temperature sensor, *PAR sensor* photosynthetically active radiation sensor, *SWC sensor* soil water content sensor. A PID (proportional–integral–derivative) controlled valve was used to stabilize pressure to a constant level

**Fig. 2:**
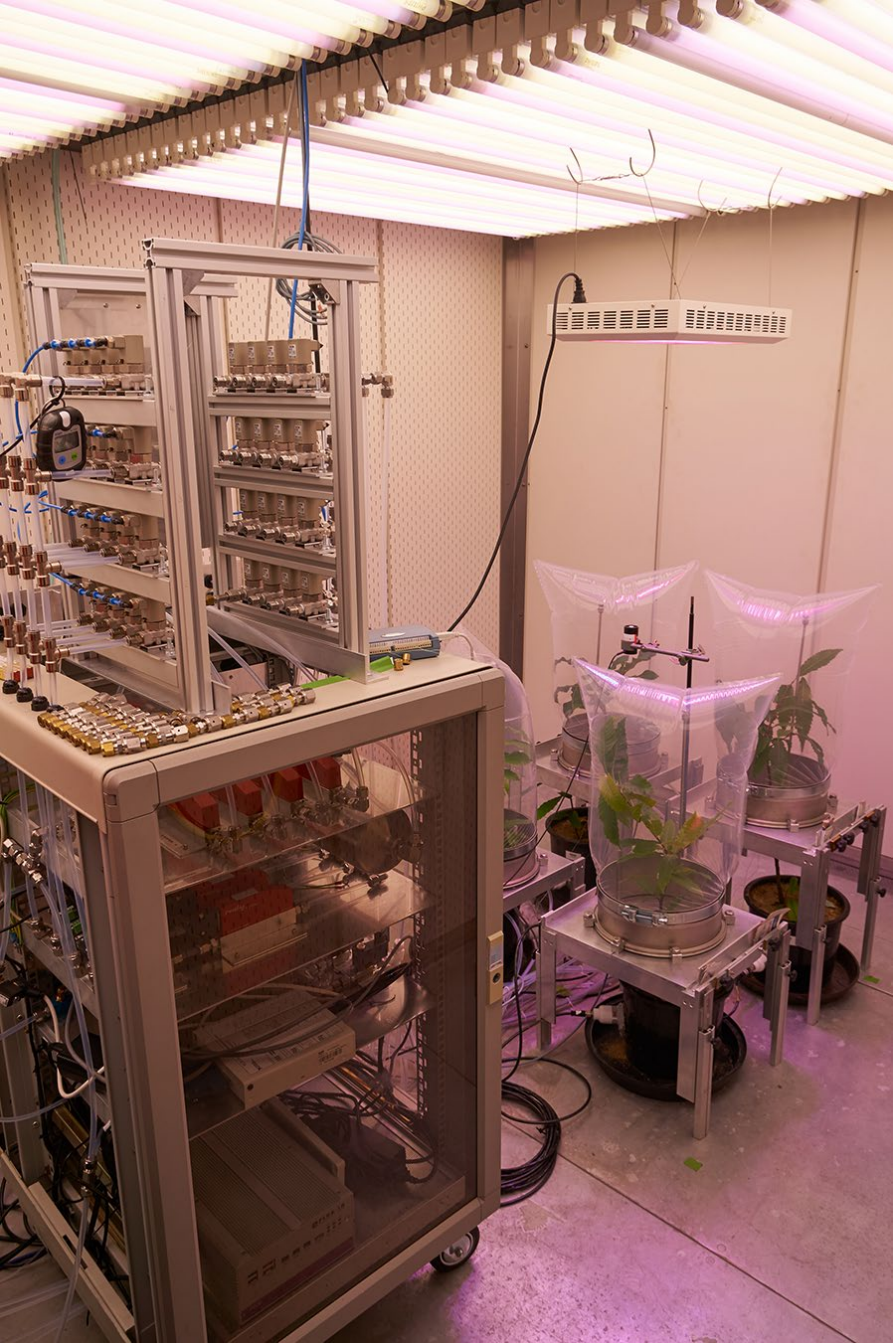
Tree DEMON set-up in a climate controlled growth chamber. *Left side* BVOC sampler unit on top of a 19 in. rack with mass flow controllers, data acquisition unit, control pc and infrared gas analyzers (not visible, located behind the rack). *Right side* four plant chamber systems with built-in trees. *Top right side* LED panel for increasing light intensity received by the plants

**Fig. 3:**
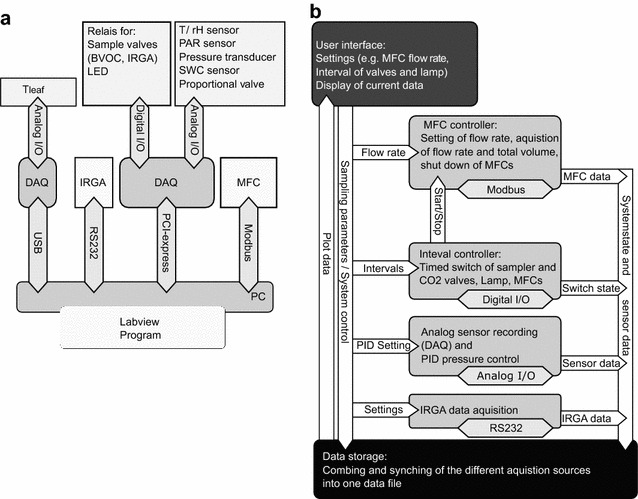
Tree DEMON software integration of different hardware. **a** Is representing the different connected data acquisition/control systems and **b** the basic software layout. *DAQ* data acquisition, *I/O* input/output, *IRGA* infrared gas analyzer, *MFC* mass flow controller, *PAR* photosynthetically active radiation sensor, *SWC* soil water content sensor, *T/rH* combined temperature and relative humidity sensor

**Fig. 4:**
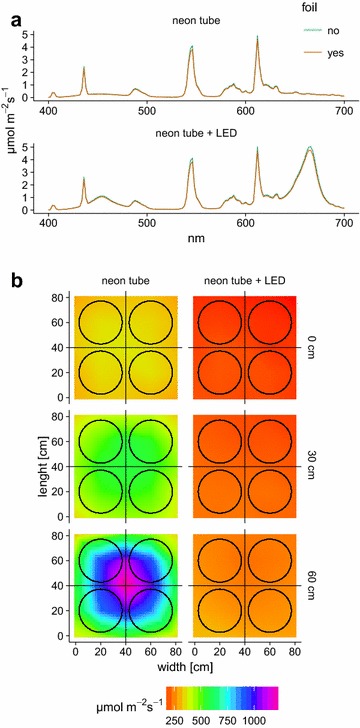
Spectral composition and distribution of the light sources. **a** Spectral response of neon tubes and neon tubes & LED together, without and with chamber foil. **b** PAR distribution of both lamp types (LED and neon tube) together and neon tube separately at three height levels (0 cm = chamber bottom plate, 30 cm = middle chamber position, 60 cm = top chamber). The plates give the interpolated mean values (bilinear) based on 5 × 5 measurement points. *Round circles* represent each plant chamber

The Tree Drought Emission MONitor (Tree DEMON) can be split into four functional units: (1) purge air supply and conditioning (Fig. 1a), (2) four dynamic plant chambers with environmental sensors (Fig. 1b), (3) BVOC sampler unit with four sample strings holding each four sample ports with adsorption tubes (Fig. 1c), and (4) CO_2_ and water vapor gas exchange unit (Fig. 1d). This set-up allows measuring BVOC and CO_2_ and water vapor gas exchange rates including chamber environment and other plant key parameters four times in parallel.

All system parts were selected for high material inertness, low gas permeability and, if possible, as industrial standard parts in order to ensure long-term maintenance. For further improving inertness almost all metal parts in contact with the chamber outlet air were chosen in grade 316 stainless steel (exceptions will be mentioned separately in the text). Since some polymers and rubber sealing can adsorb/desorb compounds [53, 54] PFA (perfluoralkoxy polymer) was used as tubing with 8 mm outer diameter. O-rings in valves and chambers consisted of FKM (fluoroelastomere) or PTFE (polytetrafluorethylene), respectively.

#### Air supply and conditioning

Chamber inlet air was conditioned in multiple steps from pressurized supply air in order to perform repeatable and reproducible gas exchange measurements (see Fig. 1a). More specifically, VOC-free air was produced by scrubbing the pressurized air with a zero air generator (AERO40LS-80, PEUS INSTRUMENTS GmbH, Gaggenau, Germany, with up to 80 l min^−1^) followed by an additional adsorption system of activated charcoal (VWR International GmbH, Darmstadt, Germany) in a 10 l stainless steel tank (RP216-1II-10-20-D, THIELMANN UCON GmbH, Hausach, Germany). Since supplied air pressure fluctuated due to the compressor intervals, pressure was stabilized with a proportional valve (PV22-20S, Aircom, Ratingen, Germany) which was controlled by a software based PID (proportional–integral–derivative) controller coupled with a pressure transducer (DRTR-ED-10V-R6B, B+B Thermo-Technik GmbH, Donaueschingen, Germany). This improved the pressure stability in the air supply for the chambers to 3 bars with a standard deviation of ±0.01 bar.

After VOC cleaning, the air was humidified by purging it through a 10 l tank filled with ultrapure water. The humidified air was further filtered through a soda lime filled 10 l tank to adsorb all ambient CO_2_. Under continuous operation the filter needed replacement after approximately two months. After air cleaning and humidification pure CO_2_ from a gas cylinder (99.995% purity, Westfalen Gas, Münster, Germany) was added to the air stream over a nozzle using a mass flow controller (SMART6 GSC, Vögtlin Instruments AG, Aesch, Switzerland, 0–200 ml_n_ min^−1^) to set the desired CO_2_ concentrations for the chamber air. Since micro fluctuation of the mass flow may lead to unsteady CO_2_ levels, a downstream 2 l stainless steel tank (Festo AG, Esslingen, Germany) was used for mixing and stabilizing the CO_2_ level. Finally, the preconditioned air was fed into the four plant chambers using four mass flow controllers (MFC) (SMART4S GSC, Vögtlin Instruments AG, Aesch, Switzerland, 0–20 l_n_ min^−1^).

#### Plant chambers and environmental sensors

The plant chamber system separated the above ground parts of the plants from the surrounding environment of the climate chamber and the root space in the pot. This set-up ensured controlled and repeatable conditions during the course of the experiments (Fig. 2). In each plant chamber air was typically exchanged with 15 l_n_ min^−1^ to reach fast steady-state conditions for the studied plants.

The top part (hood) of the chambers was built of a 25 l polyvinylidenfluorid plastic air sampling bag (Supel™ Inert Film, Sigma-Aldrich Co. LLC, St. Louis, Missouri, USA). The hood then was mounted onto a stainless steel flange (BEVAB GmbH, Bergisch Gladbach, Germany) with a diameter of 272 mm and a height of 100 mm; fixed with a metal tension band and sealed by a FKM rubber band. The resulting plant chamber volume was approximately 30 l. The lower part of the chamber consisted of two ground plates made of polished duralumin metal to separate the tree top (crown, stem) from the pot (stem, roots). Both plates were placed on a height-adjustable table. This construction was extremely solid and stable and thus allowed a fast exchange of the plants.

Air inlet and outlet to the chambers as well as the combined air temperature and relative humidity sensor (FF-IND-10V-TE1, B+B Thermo-Technik GmbH, Donaueschingen, Germany) were mounted on one of the ground plates. Leaf temperature was measured with two type K precision fine wire thermocouples (L-0044K-IEC, OMEGA ENGINEERING LTD, Northbank, Irlam, Manchester, UK) at the leaf reverse side at each tree. One soil moisture probe (SM-300, Delta-T Devices, Burwell, Cambridge, UK) was installed in each pot to measure volumetric soil water content. Photosynthetically active radiation (PAR) was measured by one PAR sensor (HOPL SKL 2620, Skye Instruments Ltd, Llandrindod Wells, Powys, UK) centered between the four chambers at mid-chamber height. Since the lamp in the climate chamber, in which the Tree DEMON system was placed for the experiments, only provided PAR around 250 µmol m^−2^ s^−1^, an additional high power multi-spectral LED lamp (Bloom Power white 360, SPLED GmbH, Flensburg, Germany) was used to increase PAR up to 550 µmol m^−2^ s^−1^.

The chamber air inlet was made of a stainless steel tube (1/4 in., closed at the end) with multiple micro nozzles (0.25 mm diameter) to generate turbulent mixing conditions within the chamber. This reduced the built-up of local concentration fields and ensured faster steady-state conditions. Both ground plates and the tree stem were sealed with a PTFE string/tape and the chamber flange were sealed by a flat FKM ring. Additionally, the chambers were operated at a slight overpressure (about 25 mbar) to prevent outside air leaking into the system. Finally, the outlet air was fed to the BVOC sample unit and CO_2_ and water vapor gas exchange unit.

#### BVOC sample unit

The BVOC enriched outlet air was sampled via a bypass with the sampler unit (Fig. 2) with four separate sampler strings. Each sampler string had four ports holding the adsorption tubes (AT). Within a port the AT was separated from the bypass by two normally closed pneumatic stainless steel valves (VXA2120M-01F-1-B, SMC Pneumatik GmbH, Gröbenzell, Germany). ATs were connected via PTFE ferrules to a VCO^®^ connector system (FITOK GmbH, Offenbach am Main, Germany) which allowed fast installation and reduced potential contamination.

At each port BVOC were sampled by drawing mass-flow-controlled outlet air (SMART4S GSC, Vögtlin Instruments AG, Aesch, Switzerland, 0–200 ml_n_ min^−1^) through the AT with a vacuum pump (GD-Thomas, Memmingen, Germany). The sample flow (standard: 150 ml_n_ min^−1^) was only active if both valves were open, so damage on the adsorption material due to a rapid pressured drop at sampling start was avoided. Furthermore, sample duration (standard: 60 min) and timing was completely customizable.

#### CO_2_ and water vapor gas exchange unit

After the sampling unit, the net photosynthesis and transpiration rate of the enclosed trees was calculated using differences of CO_2_ and water vapor in chamber inlet and outlet air monitored by a CIRAS-2 DC (PP-System, Amesbury, Massachusetts, USA) differential infrared gas analyzer system (IRGA). The IRGA measured conditioned chamber inlet air continuously at the reference channel (see Fig. 1d). Air downstream the chamber was fed via a magnetic valve manifold (E111AAV20/A/301 Fluid Concept GmbH, Karlsdorf-Neuthard, Germany) to the difference channel. This configuration allowed a subsequent monitoring of all four chambers. IRGA stability and off-set was checked by measuring reference air at both channels every 5 min. Net photosynthesis rate and transpiration rate were calculated according to von Caemmerer and Farquhar [55].

#### Software and measurement hardware

All functional units of the Tree DEMON were integrated into a LABVIEW (National Instruments, Austin, Texas, USA) based control and data measurement software (Fig. 3a, b). All sensors and relays for valve and lamp control were connected to a NI PCIe-6323 measurement card (National Instruments, Austin, Texas, USA); thermocouples were connected to a USB-TEMP measurement box (Measurement Computing, Norton, Massachusetts, USA) and MFCs were controlled over a digital MODBUS^®^ system. The IRGA was controlled and logged via a serial connection. The software controlled and recorded automatically timing and settings and signals of all MFCs, valves and lamps (Fig. 3b).

### BVOC analysis

#### Compound adsorption and thermal desorption

Inert silica coated stainless steel AT (CAMSCO, Houston, Texas, USA) with a two bed configuration of 40 mg Carbograph^®^ 5TD and 70 mg Tenax^®^ TA and a mesh size of 60/80 were used to sample and pre-concentrate emitted compounds of interest. No breakthrough of target isoprenoids compounds was detected. This was checked by placing two AT in a row and calculating the recovery rate on the first AT using sample calibration gas under standard sample settings and checking the second tube for compounds.

Compounds from the AT were transferred to the gas chromatograph (GC, see “Compound analysis” section) using an automatic thermal desorber (ATD 650, Perkin Elmer, Waltham, Massachusetts, USA). The AT was first dry purged with helium for 3 min and then the compounds were desorbed with 25 ml min^−1^ for 10 min at 280 °C with a split flow of 2 ml min^−1^. A Peltier-cooled cold trap (TurboMatrix Air Monitoring Trap™, Perkin Elmer, Waltham, Massachusetts, USA) pre-focused the desorbed compounds at −30 °C. The pre-focused sample was then injected via a transfer line (silica capillary maintained at 250 °C) with a helium carrier gas flow of 1.5 ml min^−1^ onto the separation column of the GC during ballistic heating (40 °C s^−1^) of the cold trap up to 300 °C. During the analysis of the compounds the respective AT was reconditioned with helium at 300 °C in the ATD.

#### Compound analysis

For analyzing the sample matrix, a GC/MS-FID (CLARUS^®^ SQ8, Perkin Elmer, Waltham, Massachusetts, USA) system with an Elite 5 MS column (33 m, 250 µm, 95% Methylpolysiloxane, 5% Phenyl, Perkin Elmer, Waltham, Massachusetts, USA) was used. The gas stream was split after leaving the separation column and compounds were detected using a flame ionization detector (FID) and a quadrupole electron ionization mass spectrometer in parallel. Compounds were separated by the following GC temperature program: initial temperature of 40 °C for 4 min, first ramp with 15 °C min^−1^ up to 100 °C, second ramp with 5 °C min^−1^ up to 230 °C and a 4 min hold at the end. The mass spectrometer was set to full scan mode from 33 to 330 DA and 70 eV to detect and quantify unknown compounds. The FID was set to 300 °C with a flow of 40 ml min^−1^ H_2_ and 400 ml min^−1^ zero air. Unknown substances were identified according the fragmentation patterns using the NIST database 08 [56] and if available by respective pure standards. Calibration was done by sampling a 16-component BVOC (C5–C12) gas standard (1.81–2.22 ± 0.09–0.30 nmol mol^−1^, expanded uncertainty, NPL, Teddington, Middlesex, UK) onto the AT over an extra sampler system which was identical to the one used in the Tree DEMON. Additionally, 50 ml of internal standard of Δ^2^-carene (87 ± 10.4 nmol mol^−1^, expanded uncertainty, Siad Austria GmbH, St. Pantaleon, Austria) were added onto each AT prior to air sampling. (Chromatograms of the standards can be found in Additional file 1: Fig. S1). For quantification the relative response factor (RRF) between each compound of the calibration gas and the internal standard were calculated from the FID signal of the respective compound. For compounds not present in the calibration gas, a structural equivalent RRF was used. A Level of Quantification (LOQ) of 0.004 nmol mol^−1^ was achieved and values below LOQ limit were handled as zero.

#### Emission rate calculation

Emission rate EM (nmol m^−2^ s^−1^) is determined by following equation Eq. 1 [39]:

1 $$ EM = (\chi_{out} - \chi_{in} )\,F_{M}\,A_{Leaf}^{ - 1} $$

here EM it is derived by the difference between out- and inlet BVOC mixing ratios χ_out_ and χ_in_ (nmol mol^−1^) which are multiplied by the inlet volume passing through the chamber within one second F_M_ (mol s^−1^) (determined by the mass flow controller) and divided by is the leaf area A_Leaf_ (m^2^) (see leaf area determination in the next chapter). However, it was assumed that incoming BVOC mixing ratio χ_in_ was zero due to air cleaning. Furthermore, increased water vapor induced by plant transpiration E (mol m^−2^ s^−1^) within the chamber was corrected. This is necessary since induced water vapor dilutes the BVOC concentration in the chamber and the inlet volume does not include the added water vapor, thus the mass balance has to be corrected. Inlet air cleaning and water vapor correction resulted in Eq. 2:

2 $$ EM = \chi_{out}\,F_{M}\,A_{Leaf}^{ - 1} + \chi_{out} \,E $$

Finally emissions were standardized to 30 °C and 1000 µmol m^−2^ s^−1^ for better comparability with Eq. 3 [57]:

3 $$ EM_{std} = \frac{EM}{{f_{Tl}\,f_{Q} }} $$

with the correction algorithm f_Tl_ and f_Q_ adjusting for the effects of leaf temperature and PAR, respectively (see Additional file 1: Equation S1, S2, and S3 for detailed algorithm). For comparability to other studies the emission rates were also converted into mass based emission rates (mass of emitted compound per dry mass leaf and hour in µg g_dw_^−1^ h^−1^).

### System evaluation and characterization tests

Table 1 provides an overview of conducted BVOC sampling performance tests and tests on potential chamber side effects.

**Table 1:** Performance tests of the Tree DEMON for BVOC emission studies

Test	Method	N	Settings
BVOC sampler unit repeatability and reproducibility	Sampling of Δ^2^-carene enriched air direct at chamber inlet	48 (4 sampler strings, 4 ports, three repetitions)	Sample rate 150 ml_n_ min^−1^, Sample duration: 10 min, Standard gas addition: 50 ml_n_ min^−1^ of Δ^2^-carene Chamber flow rate: 5 l_n_ min^−1^ (~0.22 nmol mol^−1^ Δ^2^-carene mixing ratio in the inlet air)
Chamber wall effects	Sampling Δ^2^-carene enriched air direct at chamber inlet and outlet simultaneously at 2 chambers	20 (2 chambers with each 2 sampler strings at inlet and outlet, 5 measurements)	See above
Residence time of compounds	Sampling system air after 1 h stop of Δ^2^-carene enrichment	12 (2 chambers with each 2 sampler strings at inlet and outlet, 3 measurements)	See above

*N* number of measurements

#### Sampler unit repeatability and reproducibility

The repeatability and reproducibility between each sampler string was evaluated by sampling Δ^2^-carene enriched air on each sample unit simultaneously. For this test, gas from the Δ^2^-carene standard instead of CO_2_ was added over a MFC (~50 ml_n_ min^−1^) into the Tree DEMON supply air. The Δ^2^-carene concentration in the enriched air was estimated to be at 0.22 nmol mol^−1^ and was sampled directly after the four inlet MFCs (5 l_n_ min^−1^) with the sampler unit. In each sampler four samples with three repetitions (N = 12 per sampler) were taken. The duration for each sampling was 10 min and a flowrate was set to 150 ml_n_ min^−1^. Afterwards the FID area count for the Δ^2^-carene was determined for each AT and was used to perform the comparison statistics. Repeatability was determined for one repetition with four samples for each sampler string by mean FID area count and its standard deviation. Repeatability for the whole sample unit was determined with the mean and its standard deviation from the mean FID area count of each sample string. Reproducibility of each sampler string was described by the mean and the standard deviation of the mean FID area count of each sampler string calculated for each of the three replications. Reproducibility for the whole sample unit was determined with the mean and its standard deviation from the mean FID area count of each sample string of all three repetitions.

#### Chamber wall effects and compound residence time

Potential chamber-wall effects were tested with the same the Δ^2^-carene addition used in the sampler repeatability and reproducibility test. Analysis of the AT was performed as described above. However, for this test the Tree DEMON sample layout was reconfigured, so that always two sample units were used for sampling inlet and outlet air of two empty chambers in parallel. The inlet and outlet chamber air was sampled five times for 10 min with a flowrate of 150 ml_n_ min^−1^ and flow rate the chamber was 5 l_n_ min^−1^.

Background air screening and residence time of the Δ^2^-carene standard was checked by taking three samples three times at in- and outlet for each chamber at 1 h before and at 1 h after feeding 50 ml_n_ min^−1^ Δ^2^-carene standard gas with an inlet air flow rate of 5 l_n_ min^−1^ into the chambers (see settings at sampler repeatability). Both tests were performed at one third of the normal chamber flow rates and at shorter sample. These settings were necessary, since with lower flow rates the Tree DEMON pressure stabilizing system regulated faster and standard gas enrichment was more stable. Additionally since no trees were installed a much higher tightness was achieved, so lower chamber flow rates could be used. Also here, the FID area counts for the Δ^2^-carene were determined for each AT and were used to perform the comparison statistics.

#### Air mixing, exchange rates and overpressure in the chambers

For reproducible measurements of gas exchange and BVOC emissions a well-mixed chamber was required in order to achieve rapidly steady-state exchange conditions and reduce local concentration fields. Air mixing of chamber was visually checked and recorded with a camera (Nex 6, Sony, Tokyo, Japan) by injecting white smoke into one chamber. The smoke was formed by passing humidified air via sulfuric acid in a smoke tube (Dräger Safety AG & Co. KGaA, Lübeck, Germany) into the middle of the chamber. Inlet air flow rate was first set to zero for a static state and switched to 10 l_n_ min^−1^ to show the dynamic mixed state.

Chambers were also tested in terms of exchange time of CO_2_ by changing CO_2_ inlet mixing ratios from 400 to 0 µmol mol^−1^ and measuring the duration until the difference channel was zero again. The chamber flow rate was set to 5 l_n_ min^−1^, which was considered as minimum chamber flow rate.

Overpressure was measured by placing a pressure sensor (LPS25H barometer sensor, Geneva, Switzerland, ST Microelectronics) into an empty chamber and measuring air pressure at 0 and 15 l_n_ min^−1^ air flow, respectively.

#### Lamp characterization and chamber PAR transmissivity

Two different types of light sources were installed and light distribution and spectral characteristics were tested, since these factors directly affects emission rates for light-dependent emitted compounds as well as photosynthesis rates. In the test set-up PAR was measured at three height levels (0/30/60 cm) above the plant chambers in a 20 cm × 20 cm grid for an 6400 cm^2^ area where all chambers fitted in. Distance from the chamber top to each light source was 65 cm for the LED and 105 cm for the climate chamber neon tubes. The spectral characteristics of the neon tubes, a 2:1 mixture of Lumilux Cool White (OSRAM, Munich, Germany) and plant lights Fluora (OSRAM, Munich, Germany), and additionally the neon tubes together with the LED were measured with a spectral radiometer (LI-1800, LI-COR, Lincoln, Nebraska, USA). Due to a slight opaqueness of the chamber material used, PAR transmissivity tests were performed. A piece of the chamber material was placed onto the spectral radiometer as well as and the extinction was measured by determining the differences of the sensors response with and without chamber material at two light levels (PAR sensor level) of 250 µmol m^−2^ s^−1^ for the neon tubes and 550 µmol m^−2^ s^−1^ for neon tube and LED, respectively. Due to the lower position of radiometer reported, which was at around 10 cm above the chamber bottom, the reported light levels are lowered to 149 and 295 µmol m^−2^ s^−1^, respectively. PAR transmissivity of the chamber was measured by placing a piece of chamber foil on the PAR sensor at light level of 187 µmol m^−2^ s^−1^.

### Case studies

The value of Tree DEMON for plant gas exchange studies was demonstrated by two case studies using sweet chestnut (*Castanea sativa* L.) trees: (1) a BVOC screening to investigate the emission composition per specimen and (2) a drought experiment to demonstrate likely impacts on CO_2_ and water vapor exchange and possibly also on BVOC emission of sweet chestnut. For both studies a total of 40 one-year-old seedlings were planted into 5 l pots with a substrate mixture of 70% sand and 30% humus already in November 2013. All pots were arranged within a greenhouse and were irrigated during wintertime by hand and in summertime by a dripping water system (Netafim Ltd, Tel Aviv, Israel) with 0.5 l per pot between two to four day intervals depending on meteorological conditions. Further, the plants were fertilized with a 0.5‰ solution (FERTY^®^ 2 and 3, Planta Düngemittel GmbH, Regenstauf, Germany) six times from May to July. For the case studies, 20 of the 40 trees were randomly selected and at a time four of those were studied in parallel.

#### BVOC screening

The BVOC screening study on the trees was performed in June 2014 after complete leaf development. Plants were checked for insect or fungal infestations and carefully cleaned from dust before installation into the Tree DEMON to ensure that no optical visible biological stressors impacted the results. 20 well-watered 2-year-old trees were sampled at 5 days from 17.06 to 25.06.2014 each twice at two light levels (250 and 550 µmol m^−2^ s^−1^) and subsequently, emission rates and compound composition were determined (detailed experiment description below).

#### Drought application

In July 2014, the effect of decreasing water availability on BVOC emission rates and CO_2_ and water vapor gas exchange was investigated on four sweet chestnut trees from the screening experiment. Trees were installed at 11.07.2014 in the afternoon and were allowed to acclimate to the climate chamber for 17 h until the first sampling. During the first two days of the experiment starting at 12.07.2014 emission of all trees were considered as non-stressed. Next, watering of two of the four specimens was stopped for six days until reaching a soil water content (SWC) of 0.04 m^3^ m^−3^. The watering of the other two trees took place between 13:00 and 15:00 with each with 250 ml of tap water. The gas exchange was assessed for each tree four times a day using the settings described below.

#### Sampling procedure and environmental settings

Plants were installed at least 12 h before the first BVOC sample to ensure acclimation to the climate chamber environment. Environmental parameters of the climate chamber (dimension h × l × w: 2.25 m × 4.45 m × 2.75 m) were set to a constant temperature of 24 °C, 50% relative humidity and a simulated 14 h day and 10 h night pattern. Light intensity of the neon tubes was controlled by a ramped program to simulate a diurnal distribution. The initial light intensity (PAR) started with 75 µmol m^−2^ s^−1^ for 1 h, followed by an increase to 150 µmol m^−2^ s^−1^ for 1 h, and a 2 h light intensity of 250 µmol m^−2^ s^−1^. From early noon to mid-afternoon an additional LED light source was used to raise the light intensity up to 550 µmol m^−2^ s^−1^ for 6 h. Light intensity in the evening was reduced with the same but reversed steps as in the morning.

BVOC sampling was conducted at two light intensities at around 250 µmol s^−1^ m^−2^ at 9:10 and 10:15 and at 550 µmol s^−1^ m^−2^ at 11:15 and 12:30 for 60 min each with a sample flow rate of 150 ml_n_ min^−1^ and a chamber air flow rate of 15 l_n_ min^−1^. Determined emission rates were standardized to 30 °C and 1000 µmol m^−2^ s^−1^ by Eq. 3.

#### Biomass assessment

Leaf area of the 20 specimens in the BVOC screening study was estimated non-invasively by measuring length and width of each leaf (screening study) since the trees were (partly) further used in the drought experiment. These parameters were converted to leaf area with a regression function fitted by the method of [58]. After the drought application, leaf area was determined with ImageJ [59] by first harvesting and then scanning the fresh leaves. Dry leaf weight was determined after drying leaves for 48 h at 60 °C. The difference between both methods was not significant tested with paired Student *t* test.

### Statistical analysis

Data processing and statistical analysis was performed with R 3.1 [60].

For assessing the repeatability and reproducibility in the system evaluation, the relative standard deviation within and between sample units was checked and an ANOVA with a post hoc Tukey’s test was performed to check for differences between the samplers. The difference between chamber in- and outlet air was checked with the paired Student’s *t* test.

For the screening study the average standardized relative emission of each tree was clustered by partitioning the relative compound information around medoids (PAM) with R package cluster [61]. The optimal cluster number was selected by the highest average silhouette size from one to ten calculated clusters.

## Results

### System evaluation

#### Tests of sampler unit

The air sampler test experiment revealed a relative standard deviation (RSD, n = 4) of the repeatability ranging between 0.74 and 2.26% using Δ^2^-carene as tracer (Table 2). Reproducibility of each sample unit showed a RSD between 0.63 and 2.15%. On average, the RSD for all sample units was 0.56% for the reproducibility and 0.62% for the single repeatability test, respectively. Further, an additional Tukey’s post hoc test on the ANOVA results for the area counts of each sampler string revealed no significant differences between each other.

**Table 2:** Repeatability and reproducibility tests

Sampler string	Repeatability (mean FID counts)	RSD (%)	N	Reproducibility (mean FID counts)	RSD (%)	N
1	3690.25	2.26	4	3706.17	0.63	3
2	3664.75	0.74	4	3664.92	0.89	3
3	3642.00	1.58	4	3674.17	0.83	3
4	3688.75	1.90	4	3661.33	2.15	3
Average	3671.44	0.62	4	3676.65	0.56	4

Tests of the sampler strings with FID counts of repeated samples of 1.5 l air with 0.22 nmol mol^−1^ Δ^2^-carene

*RSD* relative standard deviation, *N* number of repetitions

#### Chamber wall effects and compound residence time

Samples before Δ^2^-carene addition did not show any Δ^2^-carene or coeluting contaminations. Further no chamber wall effects were observed since there were no significant differences in the FID area counts between sampled Δ^2^-carene enriched air at the in- and outlet of the chambers (paired *t* test, *p* = 0.16, df = 9).

The residence time of Δ^2^-carene was less than 1 h, since samples taken 1 h after the end of internal standard addition did not show any residual Δ^2^-carene.

Additional performed blank test with preconditioned air for each chamber showed only little contamination with some compounds. These trace compound contamination occurred, however, in chromatogram windows outside the retention times for the target compounds (see also Additional file 1: Fig. S2). Possibly, the applied air filtering system was not effective enough and processes to adjust the humidity and CO_2_ concentration in the chamber air may have caused additional contamination.

#### Chamber mixing

The first test with zero chamber flow confirmed that there was almost no mixing of injected sulfuric acid particles (see Additional file 1: Fig. S3, left). After setting the air flow rate in the chamber to 10 l_n_ min^−1^ (the standard flow was 15 l_n_ min^−1^) the chamber air was already well mixed (see Additional file 1: Fig. S3, right). The mixing was completed within seconds after the onset of the chamber air flow (see Additional file 2: Video).

#### Chamber air exchange rates and tightness

The complete air exchange of one chamber took around 45 min with an inlet air flow rate of 5 l_n_ min^−1^ (one third of the standard flow rate of 15 l_n_ min^−1^), which was shown by the CO_2_ removal from a mixing ratio of 400 to 0 µmol mol^−1^ (see also Additional file 1: Fig. S4). In case of installed trees the outlet flow amounted to 50–75% of the inlet flow due to leaks in the chamber system, such as at the stem sealing. Slight overpressure in the system reduced the risk of contamination of the chamber air with outside air. The overpressure was 25 mbar in a pressure test with a chamber inlet flow rate of 15 l_n_ min^−1^. The corresponding outlet flow rate was 9.5 l_n_ min^−1^. In tests with empty chambers no target compounds were detected. Also in-chamber air CO_2_ mixing ratio (chamber: 404 µmol mol^−1^, ambient mixing ratio 400–1000 µmol mol^−1^) remained constant indicating no inflow from outside air. Even at 0 µmol mol^−1^ CO_2_ in chamber air no CO_2_ diffusion from outside was observed.

#### Lamp characterization and chamber film PAR transmissivity

The neon tube mixture showed local peaks in 436, 546, 612 and 812 nm; additionally a local increase at 490 and 650 nm was visible in Fig. 4a. The LED lamp showed its local maximal intensities at 455 and 666 nm wavelength as seen in Fig. 4a.

The upper part of the chambers was built from transparent PVDF plastic with a PAR transmissivity of 97%; therefore outside measured PAR had to be reduced by 3% for inner chamber estimates. As shown in Fig. 4a, the foil showed same reductions of PAR at all wavelengths from 400 to 700 nm. The light intensity and distribution is shown in Fig. 4b. At height levels of 0 and 30 cm we see a uniform distribution under LED + climate chamber light, whereas at 60 cm the intensity was less uniform and increased towards the center spot of the LED light.

### Case studies

#### Results of screening study

**Fig. 5:**
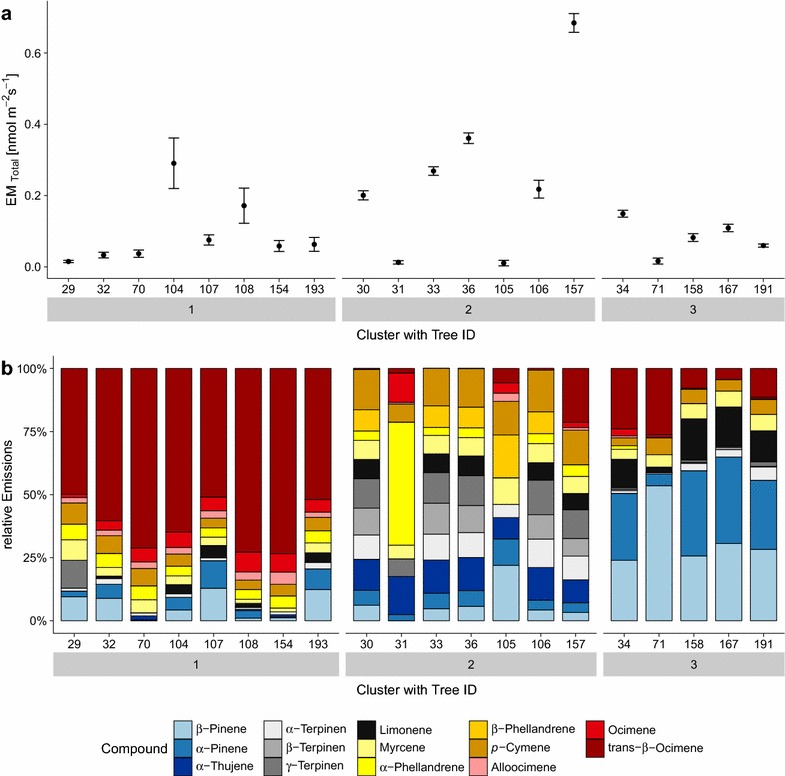
Screening study with cluster analysis. **a** Average total monoterpene emission rates of each screened sweet chestnut seedling standardized to 30 °C and 1000 µmol m^−2^ s^−1^ and corresponding cluster (1–3) assignment calculated by using PAM (Partitioning Around Medoids) method. *Error bars* represent the standard error. **b** Compound emission composition of each single tree (see ID) with corresponding cluster (1–3) calculated by PAM (see also for cluster diagnostic Additional file 1: Fig. S5)

In total 20 trees were screened in June 2014 with focus on monoterpenes and in total 15 different compounds were identified by the NIST library or by gas standards (see Fig. 5).

The standardized total monoterpene emission rate was on average 0.14 ± 0.16 nmol m^−2^ s^−1^ (0.45 ± 0.93 µg g_dw_^−1^) and ranged from almost below the detection limit [0.01 nmol m^−2^ s^−1^ (0.07 µg g_dw_^−1^ h^−1^)] up to 0.68 nmol m^−2^ s^−1^ (3.93 µg g_dw_^−1^ h^−1^; see Fig. 5a).

Analysis of the relative compound emissions by PAM clustering and silhouette width resulted in three clusters (see Fig. 5b). These clusters could be separated into a trans-β-ocimene (>50%) dominated cluster (cluster 1), an intermediate cluster with higher shares of α-/β- and γ-terpinens and α-thujene (cluster 2), and α- and β-pinene dominated (>25%) cluster (cluster 3).

#### Results of drought experiments

**Fig. 6:**
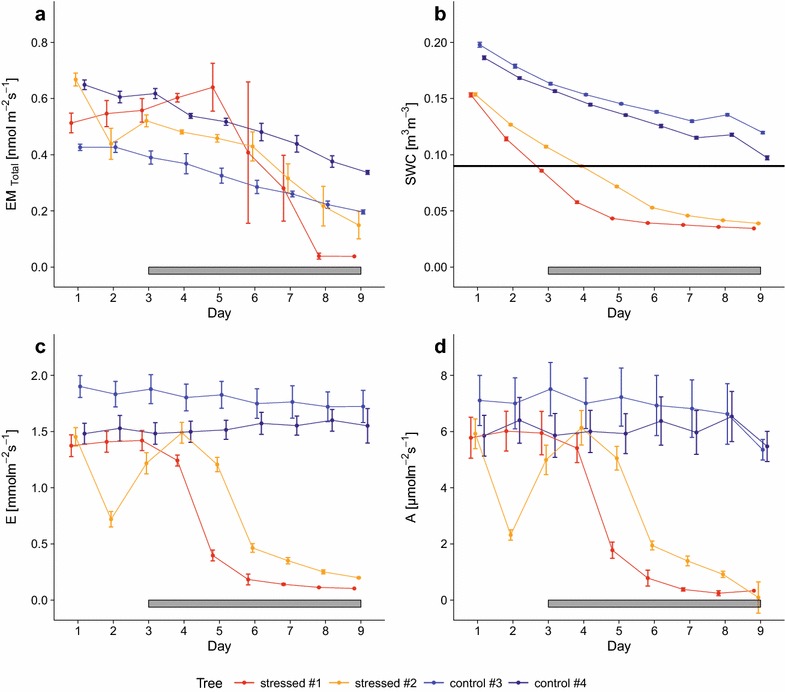
Overview on gas exchange and soil water content during the drought experiment. Gas exchange of sweet chestnut trees (#1 to #4) and soil water content during the drought stress experiment **a** mean total emission rate EM standardized to 30 °C and 1000 µmol m^−2^ s^−1^, **b** mean volumetric soil water content SWC. The horizontal black line marks the SWC value where plant gas exchange start to show a response to drought. **c** Mean transpiration rate E; **d** mean net photosynthesis rate A. *Error bars* represent the standard error of the daytime mean (N = 4). *Horizontal grey bars* indicate the day after watering was stopped for plants in the drought stress variant

In Fig. 6 daytime averaged soil water content, transpiration rate, net photosynthesis rate and BVOC emission rate for all measurements is shown for the drought application experiment. SWC served as proxy of the drought stress experienced by two trees (SWC < 0.09 m^3^ m^−3^). Non-watering of the selected trees led to a fast decline of water availability and the permanent wilting point at a SWC of 0.06 m^3^ m^−3^ was reached between day five and seven. In average an inlet concentration was measured of 403.85 ± 1.40 µmol mol^−1^ for CO_2_ and 8.47 ± 0.17 mmol mol^−1^ for water vapor at all chambers during the experiment. With respect to gas exchange, one of the control trees (#3) showed a slight decrease of the transpiration rates from 1.90 to 1.72 mmol m^−2^ s^−1^ and of photosynthesis rates from 7.10 to 5.35 µmol m^−2^ s^−1^ during the 9 days of the experiment, whereas tree #4 showed transpiration rates ranging between 1.48 and 1.60 mmol m^−2^ s^−1^ and photosynthesis rates between 6.53 and 5.47 µmol m^−2^ s^−1^. Concerning the stressed trees, #1 showed stable transpiration rates of around 1.40 mmol m^−2^ s^−1^ and photosynthesis rates of 5.95 µmol m^−2^ s^−1^ for the first three days and then during drought application a rapid decrease until the end of the experiment. Tree #2 showed a similar but later decrease of gas exchange rates during the drought application. There is no obvious explanation for the short drop of 50% in gas exchange observed on day 2 (#2).

Monoterpene emissions rate of all trees decreased during the drought experiment, despite watering of the control trees. However, drought-stressed trees showed a much stronger response to decreasing SWC. Within the first three days of the experiment, when all trees could be considered as non-stressed, emission rates EM ranged between 0.43 and 0.68 nmol m^−2^ s^−1^. At the end of the experiment the emission decreased for non-stressed trees by 50% from 0.43 to 0.20 nmol m^−2^ s^−1^ for #3 and from 0.65 to 0.34 nmol m^−2^ s^−1^ for #4, respectively. The emission rates of the stressed trees decreased from 0.52 to 0.038 nmol m^−2^ s^−1^ for #1 and 0.67–0.14 nmol m^−2^ s^−1^ for #2, respectively. However for #1, first an increase of emission was observed followed by a sharp decrease to 0.038 nmol m^−2^ s^−1^ at day 8 and 9.

## Discussion

### Tree DEMON BVOC sampling performance

Compared to other existing chamber systems (for instance two chamber system [36, 44]; three chamber system [45]) the Tree DEMON is able to investigate up to four young trees in parallel similarly to a much smaller system used for investigating leaves described by Ghirardo et al. [62]. The four chambers enable an increased sample size and allow experimental designs with two treated and two control trees investigated in parallel. The Tree DEMON takes four samples in sequence per chamber automatically, resulting in a total number of 16 samples per day, which was the optimal workload for the following chemical analysis that allowed a continuous operation over several weeks. The sample number was in the range of other automatic sorbent tube samplers, where up to 10 automatic samples [63], up to 20 automatic samples [64] and up to 24 automatic samples [37] could be taken. Performance of the sampler strings was shown with a repeatability test, in which the relative standard deviations of 0.7–2.3% were in the same range of other sample systems such as proposed by [64]. The calibration with gas standards in an identically constructed external sampler run in the laboratory compensated for likely remaining not accountable uncertainties through the sampling system, such as by dead volume and potential wall losses of valves, connectors and tubing.

The chamber supply air filtering techniques, also used by other studies [37, 44, 47, 65], allowed to maintain background VOC concentration low and free from target compounds, so an additional sampling of inlet air concentration was not necessary, thus halving the number of samples per measuring point. Additionally, the chamber air test with addition of Δ^2^-carene, serving as a proxy for monoterpenes, did not show any significant wall and memory effects of the used construction materials, reducing the number of needed blank chamber samplings. For studying other BVOC emission target compounds not measured in this study such as sesquiterpenes [63] or other aromatic species [66], it is recommended to evaluate specifically the wall and chamber effects for the selected target compounds.

### Tree DEMON system stability

For long-term investigations of treatment effects on gas exchange of plants highly reproducible settings of the assessment conditions are required. Therefore, the system was placed into a climate-controlled chamber environment and air supply for the gas exchange system was conditioned and automatically supervised to generate stable and reproducible CO_2_ and water vapor inlet air concentrations. The humidification system used in the Tree DEMON is a rather simple technique with a bubbling tank used for generating a humidification level ranging from 27 to 29% relative humidity in the inlet air due to constant pressure, temperature, and flow rates achieved with the proposed set-up. Preconditioned dry VOC free air is humidified prior to CO_2_ removal since the CO_2_ scrubber requires water to adsorb CO_2_ and to set the water content in chamber air to the desired levels. For providing the required CO_2_ amounts, pure CO_2_ was fed via a mass flow-controlled manifold to the CO_2_ depleted air stream. The highest CO_2_ scrubber efficiency was observed after 24 h conditioning time with humidified air. Over time, the water in the bubbling tank depleted and the humidity in the air downstream the humidifier may decrease slowly. Real-time supervision and control of the inlet air water content as well as CO_2_ amount ensured stable gas concentrations during the course of the experiments.

In case a more variable temperature regime with constant relative air humidity for each temperature step is needed, the proposed humidifying procedure is not ideal due to a long response time of the system. Here, other methods may be better suited e.g., a cold trap in the water saturated air stream [37] or mixing a humid air stream with a dry gas stream [67]. Another method of humidifying air has been proposed by Sun et al. [68] with refillable headspace humidifiers, where heated water generates a constant reservoir of water vapor.

If experiments in the chambers last for several days, plant physiology may change due to the artificial chamber conditions and may bias the results [69]. In case that chamber walls get in touch with parts of the plant material, even VOC emission may be induced by mechanical stress [39]. The chamber construction of the Tree DEMON ensured no or only low contact with the plant material, primarily with the PTFE sealed stem, the inlet air distribution tube inside the chamber and leaf temperature sensors. Here, only very light pressure marks were visible on the leaves, but no wounding was observed. The constructed chamber covered the whole tree top, thus investigations were more representative compared to single leaf measurements since the whole environment around the canopy is evenly controlled and not only the leaf in the chamber.

The regulation of environmental conditions in the chambers described is not as fast as for a small leaf cuvette (for instance [32, 50, 70, 71]), since air exchange rates of the chambers are smaller. Thus, the larger volume chambers are not very well suited to conduct experiments requiring fast (in the order of seconds) environmental changes. For the proposed set-up the intended CO_2_ amount in the chamber air was achieved in 5–45 min, e.g., it took 45 min with a flowrate of 5 l min^−1^) to increase the CO_2_ concentration change from 0 to 400 µmol mol^−1^. Additionally, the light regime sensed by the trees is more variable than in a single leaf chamber, since leaves have different angles and distance to the light source with self-shading effects as PAR measurements in and at different chamber positions have shown. Therefore, only a mean PAR, corrected by the chamber foil effect, was continuously recorded by placing the PAR sensor in the middle between the chambers at half of the chamber height where most of the leaf biomass was located.

### Case studies

The case studies on the sweet chestnut showed two potential applications of the Tree DEMON: (1) a BVOC emission screening study for 20 sweet chestnut trees and (2) a soil desiccation drought experiment to investigate the impact of SWC on the gas exchange. For sweet chestnut only very few emissions studies have been conducted in the past [36, 72]. Both studies showed a significant amount of monoterpene emissions, which was confirmed by this study. Yet, the total emission amount was much lower with 0.45 µg g_dw_^−1^ in our study compared to the literature values of 14.2 µg g_dw_^−1^ h^−1^ [36] and 8.41 µg g_dw_^−1^ h^−1^ from [72]. A reason for this difference could be the age of the trees (2 years) compared to adult trees used by the other studies [36, 72]. Furthermore, Pio et al. [72] used cut-off branches or leaves possibly inducing some additional emission by mechanical stress [73]. Also seasonality may affect the emission patterns and amounts as already shown for other species such as *Quercus ilex* L. [74, 75] or *Fagus sylvatica* L. [76]. Main emitted compounds from sweet chestnut trees were dominated in the study of Pio et al. [72] by β-pinene and in the study from Aydin et al. [36] by sabinene and ocimene. In our study, the emission pattern was also variable with the third identified emission cluster similar to the composition shown by Pio et al. [72]. Cluster 1, however, was *trans*-β-ocimene dominated. Paré and Tumlinson [6] reported that aphid feeding on plant induce *trans*-β-ocimene emissions. We cannot completely exclude this as a factor for the observed *trans*-β-ocimene emissions in our study since some aphid infestation was observed on a few other specimens in the greenhouse a week before the measurements started, however not on the individuals selected for our case studies.

In the soil drought experiment monoterpene emissions as well as CO_2_ and water vapor gas exchange rates declined as expected for the drought stressed plants (e.g., [70, 77]), however a slight decrease was observed for the non-stressed trees too, indicating a high sensitivity of monoterpene emissions of sweet chestnut trees to SWC. The higher standard errors for the CO_2_ and water vapor gas exchange shown in Fig. 6 are due to the two light levels within each daily measurement. The small decrease in gas exchange over time was due to the decreasing SWC, which was regulated manually and plants may have transpired more water than added.

## Conclusion

The Tree DEMON was developed and evaluated as a versatile instrument for assessing gas exchange of whole plants including BVOC emission. It allowed a high number of replicates in a short time period. Two case studies demonstrated the satisfying excellent performance of the Tree DEMON. The reliable, robust sorbent air sampling system in combination with simultaneously measured CO_2_ and water vapor gas exchange of the plants operated in a controlled environment can be used to perform mid- to long-term studies in which e.g., SWC is manipulated and confounding side effects are excluded. Furthermore, the system is easy expandable through a modular hardware and software design, so e.g., additional sample ports or more sophisticated humidity control of the chamber air can easily be implemented. The Labview software was designed to control, measure, and monitor a complete experiment to improve reproducibility and reduce potential user errors. All in one, the Tree DEMON offers an integrated solution to assess BVOC emission of plants.

## Additional files

**Additional file 1: Figure S1.** FID chromatograms of empty trap, tubes, and gas standards. **Figure S2.** FID chromatograms of internal gas standard and empty chambers. **Figure S3.** Gas mixing test. **Figure S4.** Chamber exchange rates and chamber/system tightness test with CO_2_. **Figure S5.** Cluster analysis diagnostics. **Equation S1.** General standardization algorithm. **Equation S2.** Leaf temperature correction term. **Equation S3.** Light correction term.

**Additional file 2.** Video of air mixing test in one chamber.
