# Correction to: The Tree Drought Emission MONitor (Tree DEMON), an innovative system for assessing biogenic volatile organic compounds emission from plants

**DOI:** 10.1186/s13007-017-0249-4

**Published:** 2017-11-16

**Authors:** Marvin Lüpke, Rainer Steinbrecher, Michael Leuchner, Annette Menzel

**Affiliations:** 1 Ecoclimatology, Technische Universität München, Hans-Carl-von-Carlowitz-Platz 2, 85354 Freising, Germany; 2 TUM Institute for Advanced Study, Lichtenbergstraße 2 a, 85748 Garching, Germany; 3 Department of Atmospheric Environmental Research (IMK-IFU), Institute of Meteorology and Climate Research, Karlsruhe Institute of Technology (KIT), Kreuzeckbahnstraße 19, 82467 Garmisch-Partenkirchen, Germany; 4 Present Address: Springer Science+Business Media B.V., Van Godewijckstraat 30, 3311 GX Dordrecht, The Netherlands

## Correction to: Plant Methods (2017) 13:14 10.1186/s13007-017-0166-6

After publication of this article [1], the authors noted the following error.

Due to a calculation error in the temperature term f(T_L_) of the emission standardization algorithm (Eq. 3 of the original paper), the reported emission rates have to be corrected in the text of the results as well as Fig. 5a (corrected Fig. 5) and Fig. 6a (corrected Fig. 6), and in one sentence of “Discussion”. The correction leads to overall higher emission rates, but does not affect the interpretation of the screening and drought stress case studies. Cluster analysis is not affected by this error, since relative compound shares were analyzed. Furthermore, three typos have to be corrected in the article.

The authors would like to clarify these updates in the following sections of the original article:

1. Figures
2. Figure descriptions within the text
3. Discussion section
4. Additional files

1. **Figures**

Updated figures are included with this Correction.

**Corrected Fig.** 5:

**Fig. 5:**
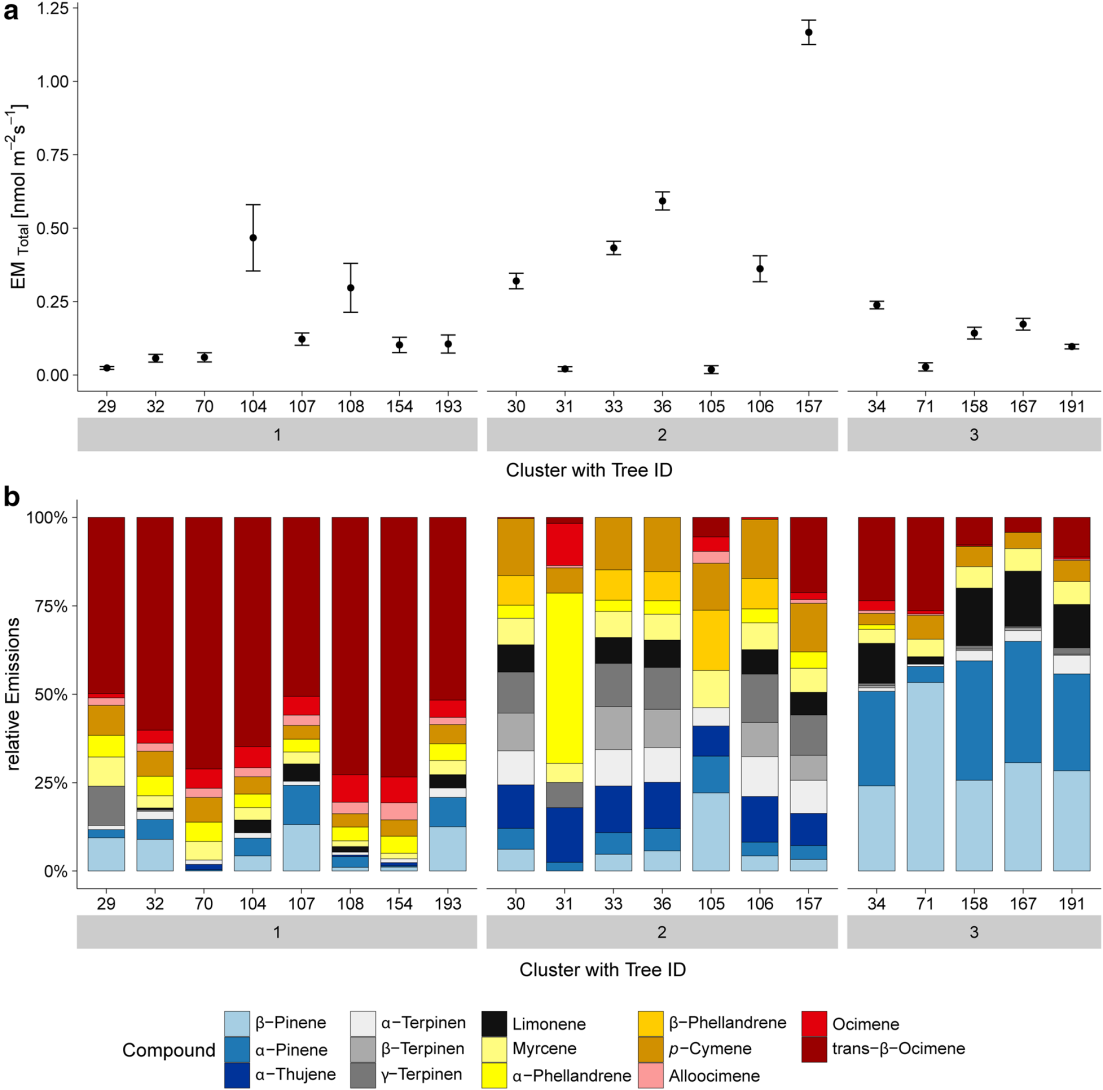
Screening study with cluster analysis. **a** Average total monoterpene emission rates of each screened sweet chestnut seedling standardized to 30 °C and 1000 µmol m^−2^ s^−1^ and corresponding cluster (1–3) assignment calculated by using PAM (partitioning around medoids) method. *Error bars* represent the standard error. **b** Compound emission composition of each single tree (see ID) with corresponding clusters (1–3) calculated by PAM (see also for cluster diagnostic Additional file 1: Fig. S5)

**Corrected Fig.** 6:

**Fig. 6:**
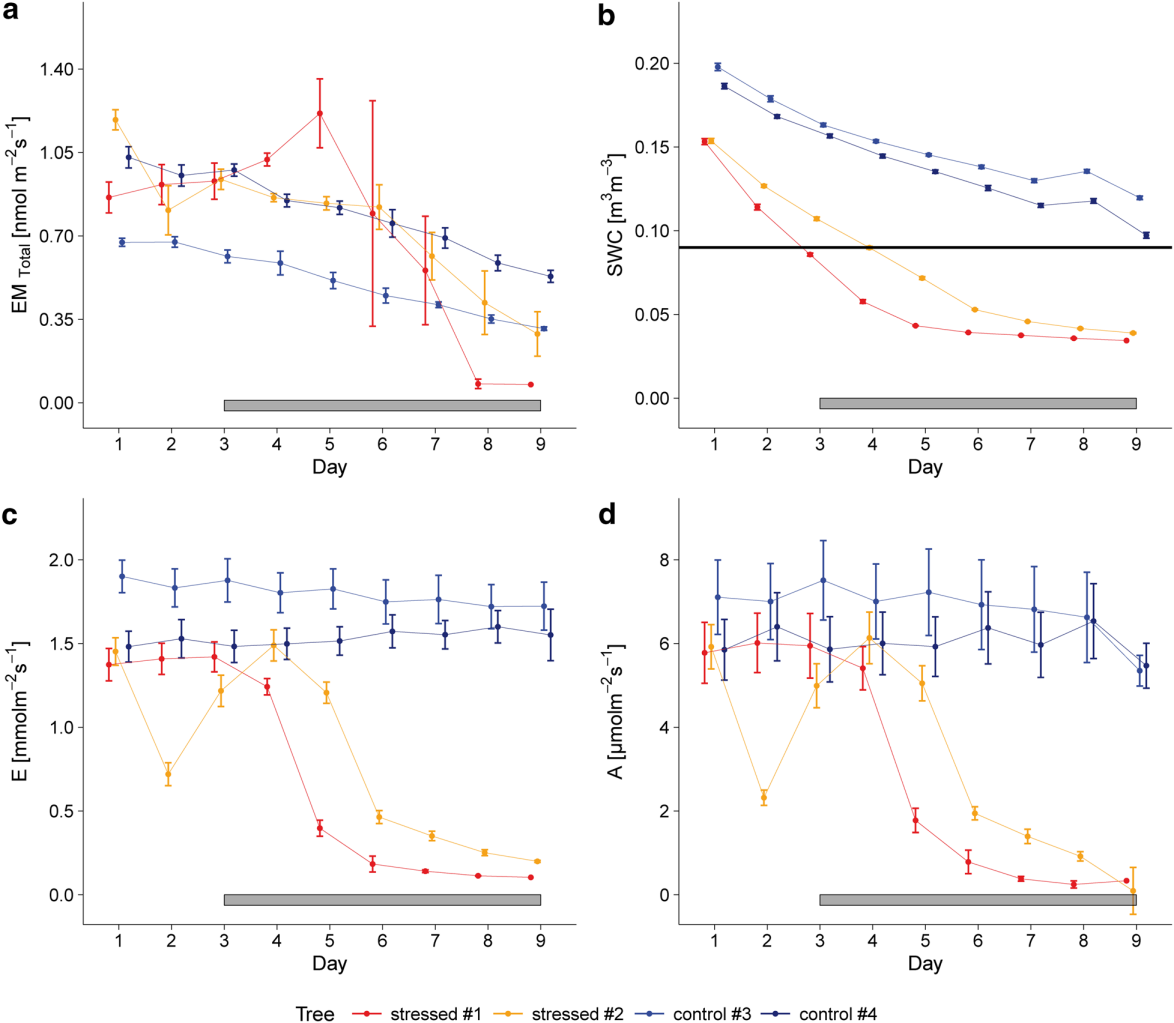
Overview on gas exchange and soil water content during the drought experiment. Gas exchange of sweet chestnut trees (#1 to #4) and soil water content during the drought stress experiment. **a** Mean total emission rate EM standardized to 30 °C and 1000 µmol m^−2^ s^−1^, **b** mean volumetric soil water content SWC. The horizontal black line marks the SWC value where plant gas exchange starts to show a response to drought. **c** Mean transpiration rate E; **d** mean net photosynthesis rate A. *Error bars* represent the standard error of the daytime mean (N = 4). *Horizontal gray bars* indicate the day after watering was stopped for plants in the drought stress variant

2. **Figure descriptions within the text**

The authors would also like to clarify the description of Fig. 5a in the original text (page 10, “Results of screening study”).

The standardized total monoterpene emission rate was on average 0.14 ± 0.16 nmol m^−2^ s^−1^ (0.45 ± 0.93 µg g_dw_^−1^) and ranged from almost below the detection limit [0.01 nmol m^−2^ s^−1^ (0.07 µg g_dw_^−1^ h^−1^)] up to 0.68 nmol m^−2^ s^−1^ (3.93 µg g_dw_^−1^ h^−1^; see Fig. 5a).

**Corrected description of Fig.** 5 **a:**

The standardized total monoterpene emission rate was on average 0.24 ± 0.27 nmol m^−2^ s^−1^ (1.40 ± 1.58 µg g_dw_^−1^) and ranged from almost below the detection limit [0.02 nmol m^−2^ s^−1^ (0.12 µg g_dw_^−1^ h^−1^)] up to 1.14 nmol m^−2^ s^−1^ (6.70 µg g_dw_^−1^ h^−1^; see Fig. 5a).

The authors would also like to clarify the description of Fig. 6a in the original text (page 12, last paragraph before “Discussion”).

Within the first three days of the experiment, when all trees could be considered as non-stressed, emission rates EM ranged between 0.43 and 0.68 nmol m^−2^ s^−1^. At the end of the experiment, the emission decreased for non-stressed trees by 50% from 0.43 to 0.20 nmol m^−2^ s^−1^ for #3 and from 0.65 to 0.34 nmol m^−2^ s^−1^ for #4, respectively. The emission rates of the stressed trees decreased from 0.52 to 0.038 nmol m^−2^ s^−1^ for #1 and 0.67 to 0.14 nmol m^−2^ s^−1^ for #2, respectively. However for #1, first an increase in emission was observed followed by a sharp decrease to 0.038 nmol m^−2^ s^−1^ at days 8 and 9.

**Corrected description of Fig.** 6 **a:**

Within the first three days of the experiment, when all trees could be considered as non-stressed, emission rates EM ranged between 0.62 and 1.20 nmol m^−2^ s^−1^. At the end of the experiment, the emission decreased for non-stressed trees by 50% from 0.67 to 0.31 nmol m^−2^ s^−1^ for #3 and from 1.04 to 0.54 nmol m^−2^ s^−1^ for #4, respectively. The emission rates of the stressed trees decreased from 0.87 to 0.08 nmol m^−2^ s^−1^ for #1 and 1.20 to 0.29 nmol m^−2^ s^−1^ for #2, respectively. However for #1, first an increase in emission was observed followed by a sharp decrease to 0.09 nmol m^−2^ s^−1^ at days 8 and 9.

3. **Discussion**

**Original text (page 14, “Case studies”)**

Yet, the total emission amount was much lower with 0.45 µg g_dw_^−1^ in our study compared to the literature values of 14.2 µg g_dw_^−1^ h^−1^ [36] and 8.41 µg g_dw_^−1^ h^−1^ from [72].

**Corrected:**

Yet, the total emission amount was much lower with 1.40 µg g_dw_^−1^ in our study compared to the literature values of 14.2 µg g_dw_^−1^ h^−1^ [36] and 8.41 µg g_dw_^−1^ h^−1^ from [72].

4. **Additional files**

**Original**:

In order to standardize the emission rate to PAR intensity of 1000 μmol m^−2^ s^−1^ and temperature of 30 °C, the algorithm in equation S1 was used (see [57] for more detailed description).

**Corrected**:

In order to standardize the emission rate to PAR intensity of 1000 μmol m^−2^ s^−1^ and temperature of 30 °C, the algorithm in equation S1 was used (see [3] for more detailed description).

In Supplement Eq. S2, one typo in the parameters is present

Original: Standard temperature: *T*_*S*_ = 314 K
Corrected: Standard temperature: *T*_*S*_ = 303.16 K

In Supplement Eq. S3, one typo in the parameters is present

Original: α = 0.0017
Corrected: α = 0.0013
