# Supplementary material for: The Tree Drought Emission MONitor (Tree DEMON), an innovative system for assessing biogenic volatile organic compounds emission from plants
Source: Plant Methods. 2017 Mar 20;13:14. doi: 10.1186/s13007-017-0166-6 (PMC5358044; doi:10.1186/s13007-017-0166-6)
Supplement: Supplementary file 1 — Additional file 1: Figure S1. FID chromatograms of empty trap, tubes, and gas standards. Figure S2. FID chromatograms of internal gas standard and empty chambers. Figure S3. Gas mixing test. Figure S4. Chamber exchange rates and chamber/system tightness test with CO2. Figure S5. Cluster analysis diagnostics. Equation S1. General standardization algorithm. Equation S2. Leaf temperature correction term. Equation S3. Light correction term. [file 13007_2017_166_MOESM1_ESM.pdf]

Supplementary online data

The Tree Drought Emission MONitor (Tree DEMON), an innovative system for assessing biogenic volatile organic compounds emission from plants

Marvin Lüpke[1], *Rainer Steinbrecher*[3], *Michael Leuchner*[1,4], *Annette Menzel*[1,2]

[1]Technische Universität München, Ecoclimatology, Hans-Carl-von-Carlowitz-Platz 2, 85354 Freising, Germany

Email: luepke@wzw.tum.de

[2]TUM Institute for Advanced Study, Lichtenbergstraße 2 a, 85748 Garching, Germany

[3]Karlsruhe Institute of Technology KIT, Institute of Meteorology and Climate Research, Department of Atmospheric Environmental Research (IMK-IFU), Kreuzeckbahnstraße 19, 82467 Garmisch-Partenkirchen, Germany

[4]now at: Springer Science+Business Media B.V., Van Godewijckstraat 30, 3311 GX Dordrecht, The Netherlands

Content

Fig. S1: FID chromatograms of empty trap, tubes, and gas standards

Fig. S2: FID chromatograms of internal gas standard and empty chambers

Fig. S3: Gas mixing test

Fig. S4 Chamber exchange rates and chamber / system tightness test with CO_2

Fig. S5 Cluster analysis diagnostics

Standardization algorithm

Equation S1: general standardization algorithm

Equation S2: leaf temperature correction term

Equation S3: light correction term

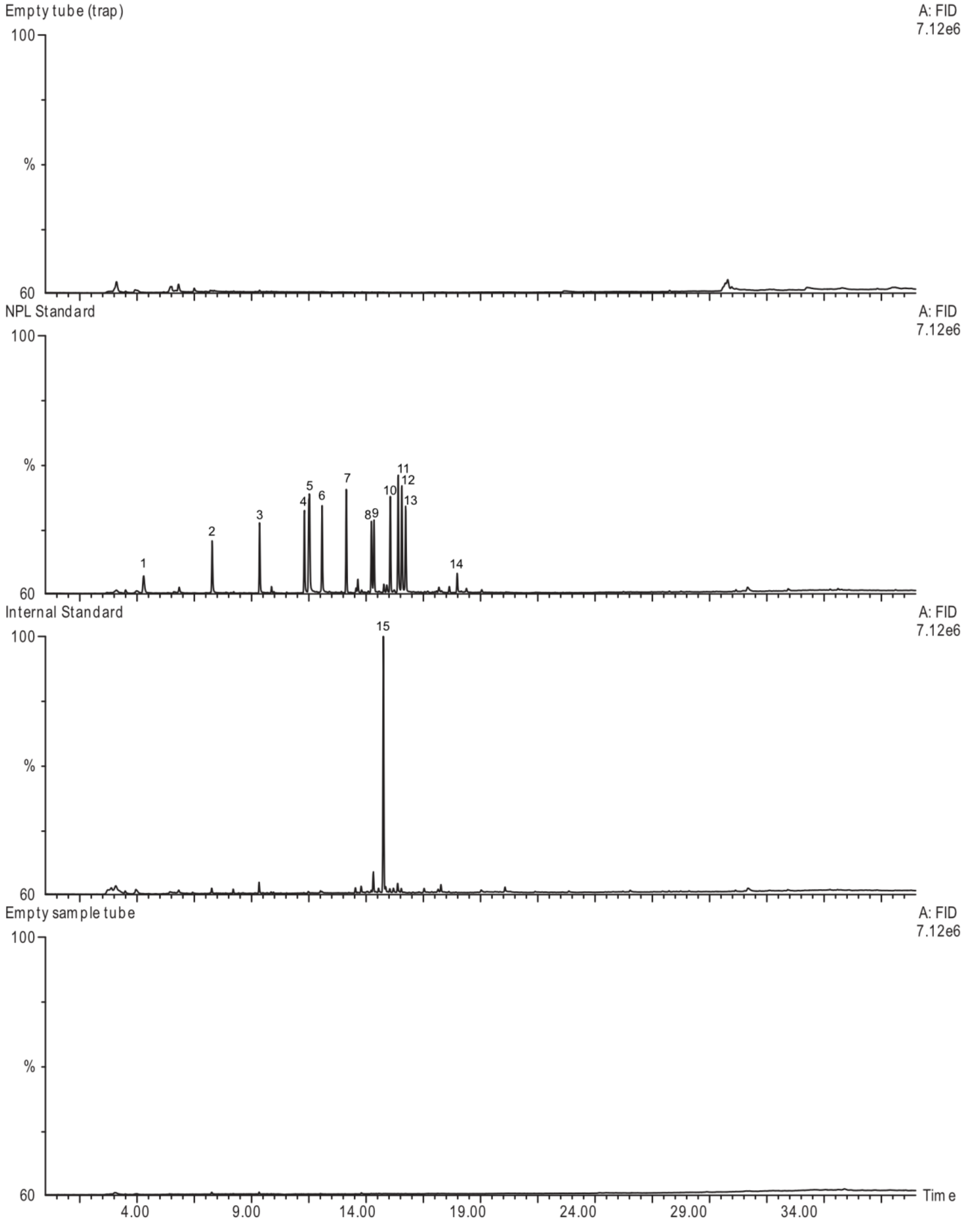

Fig. S1. FID chromatograms of an empty tube (trap); NPL gas standard with following compounds: (1) isoprene, (2) benzene, (3) toluene, (4) ethylbenzene, (5) m- and p-xylene, (6) o-xylene, (7) α-pinene, (8) myrcene, (9) β-pinene, (10) Δ^3-carene, (11) p-cymene, (12) limonene, (13) 1,8-cineole and cis-ocimene, (14) camphor; internal standard with (15) Δ^2-carene and empty sample tube.

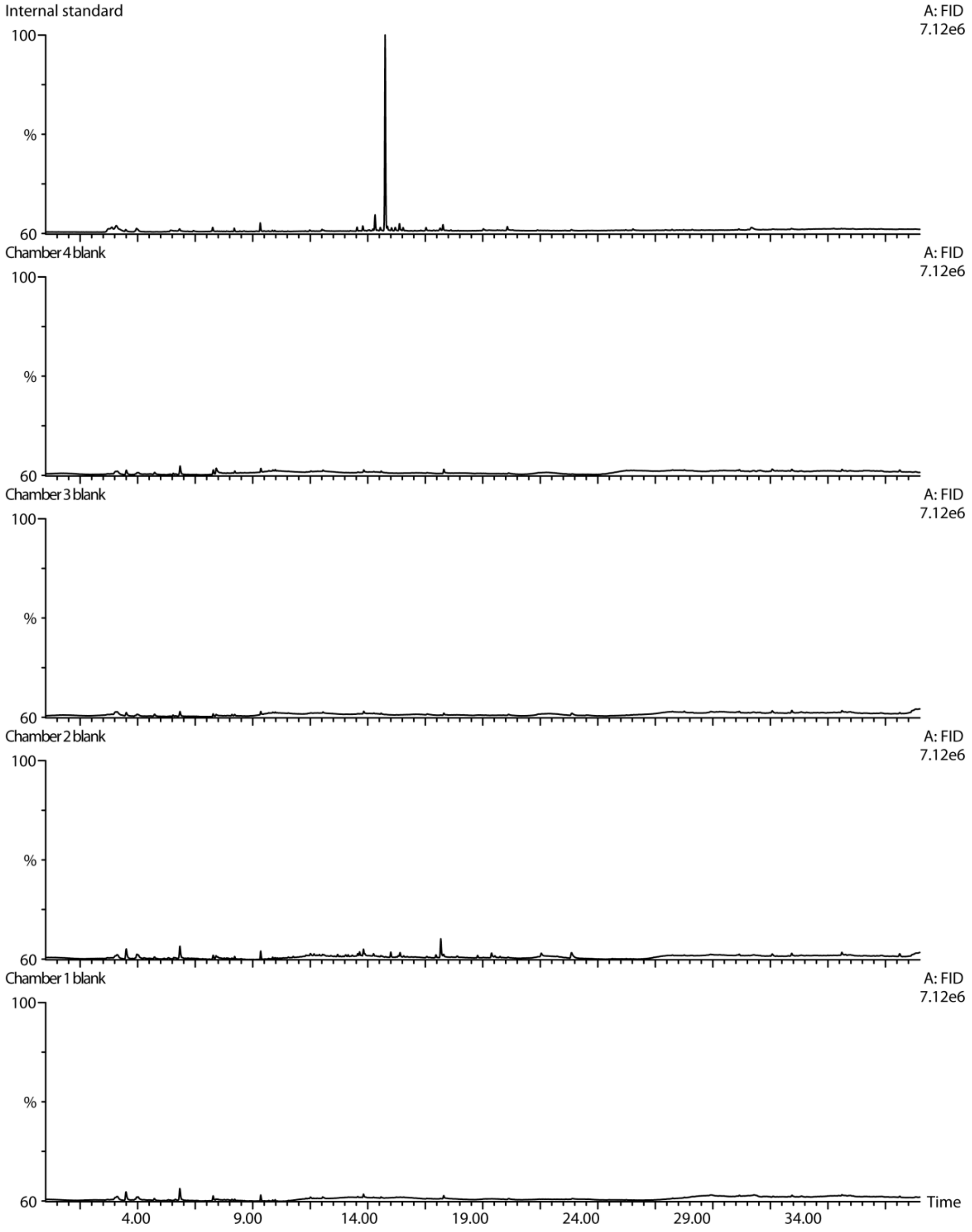

Fig. S2. FID chromatograms of internal standard and empty measurement chambers

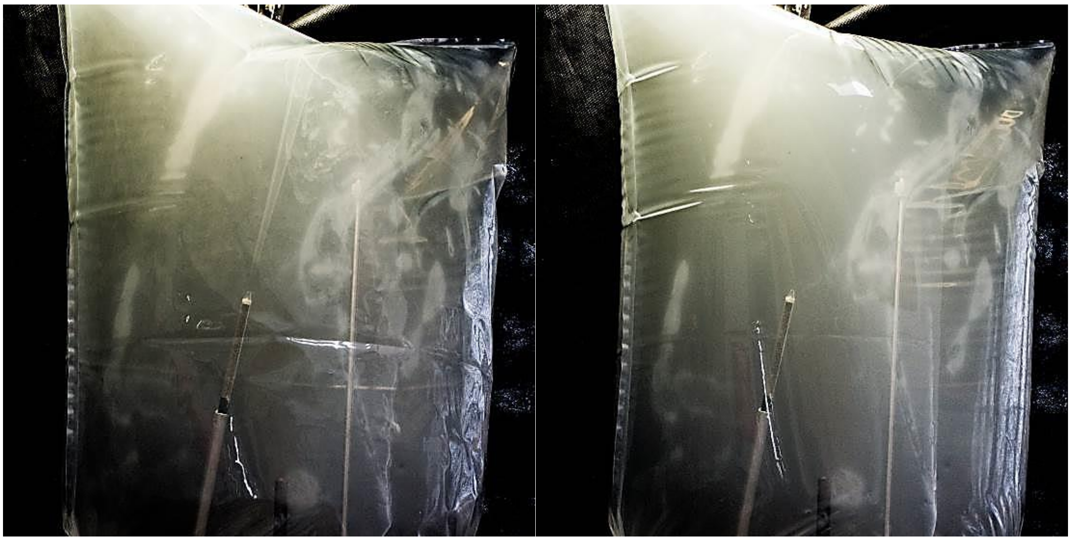

Fig. S3 Gas mixing test. Turbulent mixing within the chambers (left: no flow, right: 10 l_n min^{-1}). Black background and upward light installed to improve visibility (see video is available in Additional file 2). White areas in the left chamber represent the highly concentrated sulfuric acid particles leaving the smoke tube.

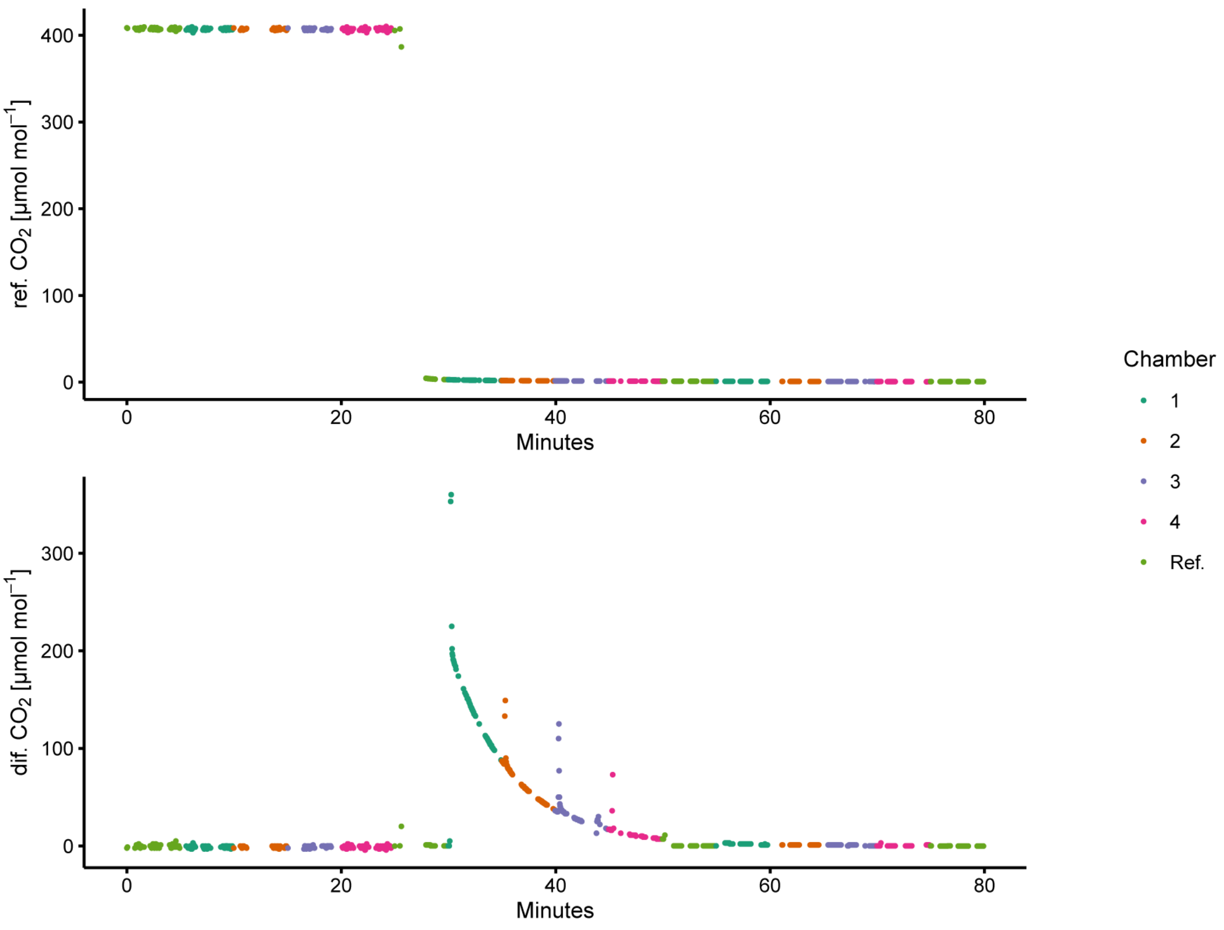

Fig. S4. Chamber exchange rates and chamber / system tightness test with the reference and difference channel measurement of CO_2 of infrared gas analyzer. Colors refer to the different chamber sampled. Test performed with a chamber flow rate of 10 l_n min^{-1}. Until around minute 25 normal CO2 addition was performed and then stopped. Here, CO2 concentration at the reference channel responded immediately, whereas the difference channel at the chamber outlet took around 25 minute to reach zero difference between both channels.

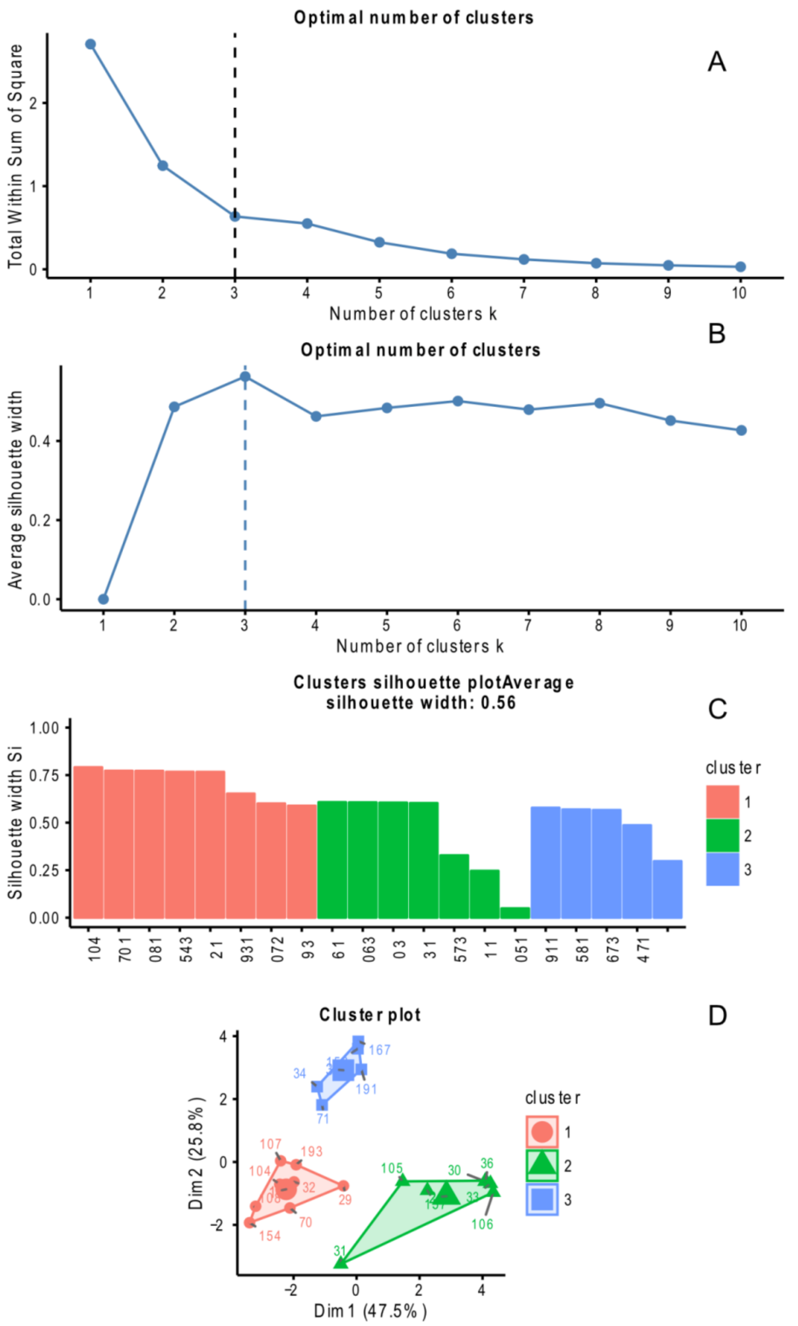

Fig. S5. PAM cluster diagnostic plots with four subplots: (A) shows the selection of optimal number of cluster by the sum of squared error (SSE) method. Here, an optimal number was found for three clusters. (B) shows the optimal number of cluster selection by the largest mean silhouette width. Also here, an optimal number of three clusters was found. In subplot (C) the silhouette plot shows how close each point in one cluster is to points in the neighboring clusters. (D) shows a cluster plot against 1st 2 principal components with a cluster number of three

Standardization algorithm

In order to standardize the emission rate to PAR intensity of 1000 µmol m^{-2} s^{-1} and temperature of 30°C the algorithm in equation S1 was used (see [57] for more detailed description)

Equation S1: $$EM_{std} = \frac{EM}{f_{Tl}\, f_Q}$$

Standardization algorithm for leaf temperature $f(T_L)$ and light f(Q)

Equation S2: $$f_{(Tl)} = \frac{exp\left[\frac{C_{T_1}(T_L - T_S)}{RT_S T_L}\right]}{1 + exp\left[\frac{C_{T_2}(T_L - T_M)}{RT_S T_L}\right]}$$

With following parameters used:

Variables defining activation and deactivation energy:

C_{T1} = 95100 J mol^{-1}

C_{T2} = 231000 J mol^{-1}

Gas constant: R = 8.314 J mol^{-1} K^{-1}

Optimum temperature: T_m= 314 K

Standard temperature: T_S= 314 K

Light dependent emission correction algorithm [57]

Equation S3: $$f(Q) = \frac{C_{L1}\alpha Q}{\sqrt{1+\alpha^2 Q^2}}$$

With scaling parameter C_{L1} = 1.26 and quantum yield α= 0.0017
